# The long-term effects of cancer survivorship on household assets

**DOI:** 10.1186/s13561-019-0253-7

**Published:** 2020-01-13

**Authors:** Tae-Young Pak, Hyungsoo Kim, Kyoung Tae Kim

**Affiliations:** 1 Department of Consumer Sciences, Sungkyunkwan University, Seoul, South Korea; 2 Department of Family Sciences, University of Kentucky, Lexington, KY US; 3 Department of Consumer Sciences, University of Alabama, Tuscaloosa, AL US

**Keywords:** Cancer treatment, Financial toxicity, Health shock, Asset depletion, Background risk, D14, I10, I31

## Abstract

**Background:**

Less is known about the impact of cancer on household assets and household financial portfolio during which cancer survivors face higher mortality risk. Economic theory predicts that cancer survivors would deplete their wealth in such a way that meets immediate financial needs for treatment and that hedges the risk of anticipated medical expenses associated with recurrence. Building upon this prediction, we examine long-term changes in household assets in response to cancer diagnosis among middle-aged and elderly Americans (age ≥ 50).

**Results:**

Using the 2000–2014 waves of the Health and Retirement Study, we estimated the household fixed effects regression that regresses household assets on time elapsed since cancer diagnosis (≤ 2 years, > 2 but ≤4 years, > 4 but ≤6 years, and > 6 but ≤8 years). Regression estimates were adjusted for demographic characteristics, general health condition, employment outcomes, and household economic attributes. Household assets were measured by total net worth as well as the amount of savings held in each asset category. The loss of household assets attributable to cancer was estimated to be $125,832 in 2015 dollars per household with a cancer patient. This change came from statistically significant reductions in investment assets, miscellaneous savings, real estate equity, and business equity, and increases in unsecured debt. We also found 17.2–28.0% increases in cash and cash-equivalent assets from + 2 years since diagnosis through the rest of the study periods. The accumulation of cash was observed for both the well-insured group (multiple coverages) and those with limited insurance (single coverage).

**Conclusions:**

The results showed evidence of both asset depletion and precautionary accumulation of liquid assets among cancer survivors, which reduces risk exposure of household financial portfolio. Our findings highlighted that household asset is an important source of liquidity to finance cancer care and to absorb the expected expenditure risk associated with cancer recurrence. We also showed that health insurance provides limited coverage of health risks associated with cancer.

## Background

The cost of cancer treatments is becoming more expensive with recent developments of immunotherapy and targeted therapeutic approaches [1]. Cancer patients pay a large portion of treatment expenses out-of-pocket (OOP) even with health insurance and suffer from lasting financial toxicity [2, 3]. Compared with individuals without a cancer history, cancer survivors carry higher medical spending for many years after initial diagnosis due to ongoing cancer care and treatments for comorbidity [4–6]. In addition, cancer incurs indirect monetary losses resulting from missed work days and reduced productivity among patients as well as their caregivers and families [4, 7, 8]. Limitations in work capability often lead to a loss of employer-provided insurance and further magnifies the financial impact of cancer.

Financial distress associated with cancer is likely more severe for older patients. Cancer incidence increases exponentially with age [9], and the prevalence of comorbid conditions makes cancer treatments more costly for the elderly [10]. Although the Medicare and the Affordable Care Act have increased access to health insurance among seniors, OOP expenditures for cancer care have diminished little by these reforms [11]. Once retired, pension income (including Social Security) and household assets are the only financial resources that can be used to finance cancer care. Since pension may not provide a sufficient cash flow, household assets would be a primary source of cancer-related expenses for older patients.

Despite a growing scholarly interest in financial toxicity of cancer care, relatively little is known about changes in household balance sheet in response to cancer diagnosis. Household asset is a more inclusive measure of financial consequences, which can capture OOP medical expenses as well as other health-related costs. Gilligan et al. [12], for instance, estimated an average of $92,098 reduction in total wealth over 4 years after diagnosis among cancer survivors aged 50 or more. Their estimate was much higher than the previous estimates of lifetime costs, which was in the range of $40,000 to $75,000 over a 15-year span [13]. By examining only OOP medical costs and omitting indirect costs and non-healthcare costs associated with cancer, prior research could have understated the financial cost of cancer. In this study, we propose an alternative approach to infer the adverse financial consequences of cancer from changes in household wealth.

Understanding the effects of cancer on household wealth is important for the following reasons. First, cancer-wealth correlation reflects the cost of cancer care as well as complex financial adjustments and its impact on survivors’ quality of life. Cancer patients with limited liquidity and credit constraint were shown to borrow against their primary residence or move out of home [14] and experience a reduction in living standards [15, 16]. They often sacrificed necessities such as clothing, food, and utilities [17, 18] and forgone medical care that treats other forms of disease [19, 20]. The high costs of cancer care and following financial adjustments were associated with poor emotional well-being and a higher risk of depression [21, 22].

Second, by examining the response of asset categories with heterogeneous characteristics, we advance our understanding of how cancer patients manage their financial assets during the post-diagnosis periods. Household assets can be used as a source of treatment expenses as well as a financial buffer against idiosyncratic health risk. A theory of risk-taking suggests that increases in background risk offset willingness to bear other forms of risk [23]. For cancer survivors receiving follow-up medical care, the risk of catastrophic medical expenditures associated with cancer recurrence constitute an important motive of stockpiling liquid financial assets. One way to achieve this adjustment might be to liquidate risky assets to pay off medical bills and keep the unused portion as emergency saving funds. We seek to answer if cancer survivors adjust their portfolio makeup in a manner that hedges the financial risk of cancer recurrence.

This study compared changes in household wealth among cancer survivors to those without a history of cancer using a nationally representative longitudinal data of Americans aged 50 or more. We estimated a dynamic model that separately identifies the immediate and delayed impacts of cancer on household wealth. The results showed that cancer patients experienced about $125,832 reduction in total net worth, and that most of this reduction was financed out of investment assets, miscellaneous saving, real estate equity, business equity, and unsecured debt. We also found 17.2–28.0% increases in liquidity (cash and cash-equivalent assets) since + 2 years after diagnosis, which had persisted through the end of the study periods (up to 8 years after onset). Further analysis highlighted that cancer survivors hold additional liquidity even in the presence of health insurance. Our finding contrasts with prior research on household financial fragility that health insurance provides enough financial protection to cancer patients [24–26].

### Literature review

Cancer survivorship is associated with a considerable financial burden on patients. A study of the 2008–2010 Medical Expenditure Panel Survey showed that a newly diagnosed cancer patient (within 1 year) aged 65 or older had an average of $16,441 annual total medical expenditures, compared with $4519 among previously diagnosed survivors [4]. A study using the cancer registry data linked to Medicare claims estimated monthly cancer-attributable costs of $1100 for lung cancer patients who received surgery and of $4809 for those who were treated with chemotherapy (in 2017 dollars) [27]. The cost estimates typically showed a U-shaped change by the phase of care: related expenses were highest in the initial year of treatment and the last year of life, and lower in the periods in-between [28]. The growth of cancer-related expenses has been leveled off beyond the acute phase but never returned to the pre-diagnosis level [29]. The long-term costs of cancer care (over a 15-year period) ranged from $65,036 and $67,806 for breast and colon cancer to $75,554 for rectal cancer ($113,615, $118,455, and $131,991 in 2018 dollars) [30]. Of the studies that concern total costs of cancer, estimates varied widely across types of treatment, cancer stage and site, population studied, and estimation method [4, 6, 28, 31].

The financial burden of cancer varies with the types and coverage of health insurance. An analysis of the 2010–2014 MarketScan data showed that an average patient with employer-provided plan had incurred about $7000–$11,000 OOP expenses over 4 years following diagnosis for a series of care that worths $100,000–$280,000 [29]. A case study from Singleterry [32] illustrated that a patient with colorectal cancer would pay about $1368 in cost-sharing after having the majority of the total costs ($123,057) covered by Medicare and Medigap plans. In the case of a patient with breast cancer and having employer-sponsored insurance, the OOP burden on a patient is projected to be $3975 out of $144,193. Evidence from the 2002–2012 waves of the Health and Retirement Study showed that higher OOP costs were associated with less generous health insurance; mean annual OOP spending incurred after a cancer diagnosis were $2116 among those on Medicaid, $2367 among those with coverage from the Veterans Health Administration, $5976 for those insured by a Medicare health maintenance organization, $5492 among those with employer-provided coverage, $5670 among those with Medigap, and $8115 among those with a traditional fee-for-service Medicare coverage but without supplemental coverage [33].

Cancer incurs direct medical costs as well as indirect costs resulting from productivity loss. Examples of indirect costs include time spent receiving medical care, time lost from work or other usual activities, lost productivity due to premature death, and lost household productivity for caregiving [34]. A study of cancer survivors in Canada found that the onset of cancer is associated with an average of 10% reduction in earnings and 5 percentage points decline in the probability of working [35]. The US study using data from the 2008–2011 Medical Expenditure Panel Survey (MEPS) showed that nearly one in three survivors had limitations in usual daily activities outside of work, and one in four felt less productive at work [36]. Among breast cancer survivors, the onset of cancer was associated with a significant increase in absenteeism and disability days [37] in a group with comprehensive health insurance as well as in a group with less coverage [38]. Mean annual indirect costs ranged from a low of $380 for patients with prostate cancer [39] to as much as $16,472 for patients with breast cancer [37].

Cancer survivors are at risk for financial hardship, as evidenced by the accumulation of medical debt and a higher chance of default. Approximately one-third of cancer survivors reported the use of consumer credits post-treatments, with half of them incurring obligations of $10,000 or more [40]. Among breast cancer survivors, 23% went into debt for treatment-related expenses and held a mean debt of $26,860 [41]. Using consumer credits to finance cancer care leaves a long-lasting impact on household balance sheets that persists more than 4 years [14]. Cancer survivors often go bankrupt as a result of their treatment costs. A cancer diagnosis was associated with 2.65 times higher chance of filing for bankruptcy in the state of Washington [42]. Patients with more toxic cancers, such as lung, colorectal, and thyroid cancers, were more likely to declare bankruptcy as compared to those with other types. In a study of US Bankruptcy Court data, medical bankruptcies accounted for more than 50% of all bankruptcy cases [43]. Besides, several studies noted that a large number of cancer survivors had to cut down spending on basic necessities [44] and exhaust savings to pay medical bills [3, 14, 18, 45].

The financial burden of cancer care expense is associated with psychological distress and subjective ill-being. A study of Hispanic women with breast or gynecological cancer showed that 68% of them had medical cost concerns, 47% had wage concerns, and 49% was subject to financial stress [46]. The cancer survivors with medical cost concerns and worries were more likely to report depressive symptoms and less likely to report positive evaluation of functional, emotional, and physical well-being. The subsequent study by Chino et al. [21] used the patient satisfaction questionnaire and found a significant association between high financial burden and dissatisfaction with general health care, quality of cancer care delivery, and financial aspects of health care. In a study of patients with stage III colorectal cancer, complications after surgery were shown to exacerbate financial burden on patients and increase worry about finances [17].

Beyond quality of life, financial distress has been shown to change decision making regarding treatment. Examples of changes in treatment-related decision making include treatment non-adherence and forgoing/delaying suggested cancer care. Markman and Luce [47] found that 11% of cancer patients considered treatment cost when choosing a course of treatments and 9% forgone the suggested treatment course due to cost-related concerns. Zafar et al. [3] showed that insured cancer patients seeking copayment assistance were more likely to replace suggested medications with over-the-counter drugs in an attempt to reduce OOP expenses. Among cancer survivors faced with financial hardship, about 5–20% cut spending on medical services unrelated to cancer [19].

A review of the cost estimates for cancer care in the United States found significant heterogeneity across the types of tumor, baseline data, study methods, and population studied [13]. Sources of cost data included insurance claims, medical bills, hospital discharge data, and self-reports [28, 34]. The limitation of the prior approach is that (a) calculation of indirect costs builds upon strong assumptions, and (b) existing insurance claim data does not provide information on those not having health insurance. The alternative approach, which has been followed in some prior research, is to infer the cost of cancer diagnosis from changes in household assets before and after diagnosis. For instance, Lee and Kim [48] examined the impact of new health events and existing health conditions on wealth depletion in later life. Kim and Lee [49] linked compounded health problems to wealth changes and found that wealth depletion was most pronounced for those with a combination of heart disease and diabetes. Similar approaches have been used in Spicer et al. [50], Poterba et al. [26], and Wu [51]. Our study extends this line of research by estimating long-term changes in wealth following a cancer diagnosis.

## Methods

### Data description

The sample is drawn from the 2000–2014 waves of the Health and Retirement Study (HRS). The HRS is a nationally representative survey of the noninstitutionalized population aged 50 years and older and their spouses [52]. The survey has been fielded every other year since 1992 with a goal of tracking lifestyle changes of American seniors. Information collected ranges from demographic, socioeconomic, and health characteristics of individual respondents to their household assets and income.

We apply the following sample selection criteria. First, we eliminate respondents younger than age 50 from the sample. Individuals dropped at this stage are predominantly female spouses who are included in the study as a spouse of the age-eligible householder. Second, we drop the Mid Baby Boomer cohort (born in 1954–1959). This cohort has been tracked only for three waves and does not provide enough observations for panel data analysis with lagged regressors. Third, we delete households in which one or more spouses died from cancer or those reported multiple cancer diagnoses during the study period. These groups would exhibit a different pattern of asset depletion for reasons unrelated to cancer, such as bequest and donation at the end of life, and confound regression estimates for the cancer effect. Lastly, we delete respondents with only one observation in order for our estimation strategy to exploit within-person variations. The final sample is an unbalanced panel of 68,634 observations for 16,451 respondents (1354 cancer survivors and 15,097 individuals without a history of cancer) and 10,933 households.

### Measures of household assets

The HRS gathered information on household wealth and income through the survey of a financial respondent in each household. Our summary measure of household wealth is total net worth - the sum of cash and cash-equivalent assets, stocks, bonds, miscellaneous savings, retirement savings, housing, vehicles, real estate, and private business, minus unsecured debt. All monetary values are converted to 2015 dollars using the consumer price index for all urban consumers (CPI-U).

While aggregate measures show overall changes in household assets, they would not reveal the different pattern of depletion in assets with heterogeneous characteristics. For instance, cancer patients may use liquid assets for treatments in the acute phase and exhaust non-financial assets, such as residence and real estate, for subsequent treatments. Some patients would reallocate their illiquid assets to cash and cash-equivalents to cope with a chance of catastrophic medical expenses associated with recurrent cancer. To examine these channels, in the second phase of analysis we examine the following asset categories separately: (a) cash and cash-equivalents, (b) stocks and bonds, (c) miscellaneous savings, (d) individual retirement accounts, (e) housing equity, (f) vehicles, (g) real estate equity, (h) private business, and (i) unsecured debt. The detailed description of asset variables is presented in Table 1.

**Table 1:** Measures of household asset

	Definition
Cash and cash equivalents	Checking accounts, savings accounts, money market accounts, CD, government savings bonds, and T-bills
Stocks	Stocks, mutual funds, and investment trusts
Bonds	Bonds and bond funds
Miscellaneous savings	Jewelry, money owed by others, a collection for investment purposes, rights in a trust or estate, or an annuity
Retirement savings	IRA and Keogh accounts (net value)
Housing equity	Primary and secondary residence (net value)
Vehicles	All vehicles owned by a household (net value)
Real estate equity	Real estate other than home (net value)
Business equity	Private farm or business (net value)
Unsecured debts	Credit card balances, medical debts, life insurance policy loans, loans from relatives

### Measure of cancer diagnosis

Respondents with a newly diagnosed cancer are identified through the following survey question, “*Since the previous interview, has a doctor ever told you that you have cancer or a malignant tumor, excluding minor skin cancers?*.” Responses to this question are converted to a household-level cancer indicator, which takes on one for households with a spouse newly diagnosed with cancer and zero otherwise. The fraction of married couples with cancer diagnosis ranges from 3.0 to 3.8% of the sample in each survey wave. Approximately 8.2% of our final sample exhibit a history of cancer diagnosis during the study period.

### Empirical specification

The regression model for household *i* in time period *t* takes the following form,

$$ {y}_{it}=\psi +{\beta}_0{c}_{it}+{\beta}_1{c}_{it-1}+{\beta}_2{c}_{it-2}+{\beta}_3{c}_{it-3}+{X}_{it}\lambda +{v}_i+{v}_t+{\varepsilon}_{it},(1) $$

where *y*_*it*_ is a measure of household asset, *c*_*it*_ is binary indicator for cancer onset between period *t* − 1 and *t*; *X*_*it*_ is a vector of covariates; and *v*_*t*_ is a common time trend in wealth outcome (e.g., business cycle) that will be captured by year-of-survey dummies. All regressions include three lags of the cancer indicator (*c*_*it* − 1_, *c*_*it* − 2_, and *c*_*it* − 3_) to capture the lasting effects of cancer on household assets.^1^ Since we include lagged regressors, the outcome variable and covariates (*y*_*it*_, *X*_*it*_, and *v*_*t*_) are based on five survey waves from 2006 to 2014.

The error term consists of the *i.i.d.* random error (*ε*_*it*_) and a household-specific time-invariant factor that captures unobserved household heterogeneity (*v*_*i*_). To account for the potential confounding effects of *v*_*i*_, we estimate a household fixed effects (FE) model. This approach differences out *v*_*i*_ and uses only the within-household variation to estimate regression parameters. The parameters of interest (*β*_0_, *β*_1_, *β*_2_, and *β*_3_) capture between-survey changes in household wealth due to the onset of cancer in the current and previous periods.

A covariate vector *X*_*it*_ includes time-varying characteristics of respondents or households that might be correlated with household assets. These variables include age and age squared, marital status, number of children, self-rated physical health, labor force participation, health insurance ownership, annual household income, transfer of financial resources between adult children and respondents, and Census division of residence. The age effect is modeled in a quadratic form to allow household wealth decumulate at an increasing rate over age. Respondents’ overall health condition is captured by a five-category self-rated health (poor, fair, good, very good, and excellent). For marital status, we include dummies for married, divorced or separated, and widowed in reference to never-married. Labor force participation is categorized into fully employed, partially employed, unemployed, retired, and not in the labor force. Health insurance coverage is coded with three exclusive categories: (a) no insurance, (b) single health insurance coverage or two or more government-provided plans (Medicaid, Medicare, both Medicaid and Medicare, veteran’s insurance, a single private plan, or coverage through spouse’s plan), and (c) multiple health insurance coverages or employer-provided plan.

To account for high skewness in outcome variables, the income and wealth variables (household asset, household income, and intergenerational transfers) are transformed by the log-modulus transformation [53]. This transformation takes a log on the absolute value of *y*_*it*_ plus 1 and multiplies this quantity by − 1 if the original value is negative. The formula can be expressed as, sign(*y*_*it*_) ∙ log(∣*x* ∣  + 1). This approach has advantages over a naïve log-transformation in that it preserves the location of zero values and spread out negative values across the same logarithmic scale on a negative domain. Interpretation of marginal effects remains unchanged.

## Results

Table 2 presents average descriptive statistics for the full sample and separately for a non-cancer group and a cancer survivor group. The last three columns present cancer survivors’ characteristic for pre-diagnosis, initial phase of treatment (≤ 2 years after diagnosis), and continuing phase of treatment (> 2 years after diagnosis). The sample is about 69.6 years old, 58.7% female, and 65.3% married. Approximately 92.4% reported one or more health insurance coverage (including Medicaid and Medicare), and 70.9% assessed their overall health good or better. Comparing respondents by cancer history shows that cancer has been more prevalent in males (46.6% vs 40.5%; *P* < .01), older respondents (70.0 vs 69.5; *P* < .01), retirees (71.4% vs 64.8%; *P* < .01), and households with more assets ($595,873 vs $524,858; *P* < .01) and income ($72,024 vs $69,586; *P* < .01). The mean total net worth was reduced from a pre-diagnosis average of $659,537 to $608,993 in the initial phase and to $515,604 in the continuing phase. The liquidation pattern is more pronounced for non-housing illiquid assets, such as real estate equity and business equity. The amount held in cash and cash-equivalent assets is higher in the continuing phase, relative to the initial phase ($58,477 vs $54,078; *P* < .01).

**Table 2:** Average descriptive statistics

	Full sample (*N* = 68,634)	No history of cancer (*N* = 58,593)	Cancer survivor (*N* = 10,041)	Before diagnosis (*N* = 4121)	Initial phase (*N* = 2279)	Continuing phase (*N* = 3641)
Individual characteristics						
Age	69.6	69.5	70.0			
Female^a^	58.7	59.5	53.4			
Married^a^	65.3	63.2	77.8			
Separated or divorced^a^	12.6	13.3	8.5			
Widowed^a^	18.5	19.6	12.0			
Never married^a^	3.5	3.8	1.8			
Number of children	3.2	3.2	3.2			
Self-rated health: good or better^a^	70.9	71.1	70.0			
No health insurance^a^	7.6	8.0	5.6			
Single health insurance^a^	46.2	46.6	43.7			
Multiple health insurance^a^	46.2	45.4	50.8			
Fully employed^a^	20.2	20.7	17.0			
Partially employed^a^	4.5	4.7	3.3			
Unemployed^a^	1.8	1.8	1.5			
Retired^a^	65.8	64.8	71.4			
Not in the labor force^a^	7.7	7.9	6.7			
Household income and wealth^b^						
Total income	69,943	69,586	72,024	80,919	71,599	62,222
Total net worth	535,247	524,858	595,873	659,537	608,993	515,604
Cash and cash equivalents	49,987	48,764	57,124	57,613	54,078	58,477
Stocks and bonds	88,705	90,327	79,240	84,887	79,624	72,609
Miscellaneous savings	14,845	14,091	19,240	23,623	17,917	15,109
Retirement savings	79,319	76,675	94,749	96,936	96,191	91,372
Housing equity	184,538	180,423	208,555	223,915	204,507	193,702
Vehicles	16,693	16,349	18,702	20,035	18,023	17,617
Real estate equity	53,127	52,553	56,475	69,481	66,706	35,351
Business equity	52,970	50,544	67,128	89,373	78,155	35,049
Unsecured debts	4938	4869	5340	6325	6208	3681

Initial phase represents the first HRS survey followed by a cancer diagnosis (≤ 2 years after diagnosis). Continuing phase represents all subsequent periods after the initial phase (> 2 years but ≤ 8 years after diagnosis)

^a^Individual characteristics (excluding age and number of children) represent percentage figures

^b^Household income and wealth are adjusted to 2015 dollars using the Consumer Price Index for all urban consumers

Table 3 presents estimates from the regression of total net worth on cancer and covariates. We first estimate a null model (column 1) and then add additional controls to evaluate the robustness of our results (columns 2 and 3).

**Table 3:** Cancer effects on household wealth (*N* = 68,634)

Response:	Log (Total NW)	Log (Total NW )	Log (Total NW)
	(1)	(2)	(3)
*β*_0_: Cancer *t*	−0.260***	− 0.288***	− 0.268***
	(0.088)	(0.087)	(0.087)
*β*_1_: Cancer *t* − 1	−0.152	−0.179*	− 0.173*
	(0.094)	(0.094)	(0.094)
*β*_2_: Cancer *t* − 2	−0.132	− 0.141	− 0.143
	(0.088)	(0.088)	(0.088)
*β*_3_: Cancer *t* − 3	−0.151	−0.158*	− 0.154*
	(0.093)	(0.093)	(0.093)
Age		0.169***	0.148***
		(0.033)	(0.033)
Age squared (/100)		−0.109***	−0.094***
		(0.023)	(0.023)
Married		1.089***	1.011***
		(0.324)	(0.322)
Separated or divorced		−0.429	−0.411
		(0.331)	(0.330)
Widowed		0.517	0.487
		(0.324)	(0.323)
Number of children		−0.020	−0.021
		(0.042)	(0.042)
SR health: fair			0.358***
			(0.081)
SR health: good			0.408***
			(0.080)
SR health: very good			0.457***
			(0.083)
SR health: excellent			0.523***
			(0.093)
Single health insurance			0.108
			(0.109)
Multiple health insurance			0.273**
			(0.110)
Fully employed			−0.070
			(0.089)
Partially employed			0.080
			(0.114)
Unemployed			−0.194
			(0.176)
Retired			0.048
			(0.072)
Log(HH income)			0.141***
			(0.021)

Robust standard errors in parentheses. Subscript *t*-1, *t*-2, and *t*-3 represent variables measured in one, two, and three survey periods earlier (2, 4, and 6 years prior to the measurement of wealth). Total NW represents total net worth. Regressions control for the log of intergenerational transfers, year-of-survey dummies, and Census division fixed effects. *** *p* < 0.01; ** *p* < 0.05; * *p* < 0.10

All three columns show that the onset of cancer was followed by immediate reductions in total net worth. The coefficient estimates on cancer at *t* − 1 carry a negative sign and reject the null hypothesis of non-significance at the 1% level. In our preferred specification (column 3), the coefficient estimate on the immediate cancer effect is − 0.268, with a 95% CI ranging from − 0.439 to − 0.097. This estimate corresponds to a 23.5% (=1 − *e*^−0.268^) reduction or a $125,832 loss ( = *$*535,247 × (1 − *e*^−0.268^)) in total net worth, evaluated at the sample mean. The estimated cancer effects for the subsequent periods maintain a negative sign but are not different from zero at the 5% level. In all specifications, asset depletion is more pronounced for the acute phase of treatment. Coefficient estimates for the covariates show that household assets increase with being married, self-reported health, health insurance, and total income, but decreases with cancer diagnosis.

Table 4 presents estimates for associations between cancer diagnosis and the amounts held in each asset category. Regressions control for all explanatory variables, including year-of-survey fixed effects and Census division dummies. The estimates for covariates are omitted for brevity.

**Table 4:** Cancer effects on household wealth, by asset categories (*N* = 68,634)

Response:	Log (cash)	Log (stocks+bonds)	Log (miscellaneous)	Log (retirement)	Log (housing)	Log (vehicles)	Log (estate)	Log (business)	Log (unsecured debt)
	(1)	(2)	(3)	(4)	(5)	(6)	(7)	(8)	(9)
*β*_0_: Cancer *t*	−0.021	−0.170**	− 0.163**	0.002	− 0.063	0.017	−0.167**	− 0.078	0.236***
	(0.059)	(0.081)	(0.075)	(0.074)	(0.087)	(0.052)	(0.067)	(0.055)	(0.076)
*β*_1_: Cancer *t* − 1	0.247***	−0.369***	0.002	0.020	−0.075	0.012	−0.072	−0.145**	0.282***
	(0.061)	(0.087)	(0.087)	(0.081)	(0.096)	(0.060)	(0.075)	(0.061)	(0.081)
*β*_2_: Cancer *t* − 2	0.184***	−0.401***	− 0.017	− 0.075	− 0.053	0.017	0.008	− 0.175***	0.171**
	(0.068)	(0.092)	(0.090)	(0.088)	(0.094)	(0.061)	(0.078)	(0.063)	(0.082)
*β*_3_: Cancer *t* − 3	0.159**	−0.058	− 0.175*	− 0.028	− 0.066	0.123*	− 0.114	−0.011	0.170**
	(0.073)	(0.090)	(0.090)	(0.092)	(0.092)	(0.064)	(0.076)	(0.063)	(0.081)

Robust standard errors in parentheses. Subscript *t*-1, *t*-2, and *t*-3 represent variables measured in one, two, and three survey periods earlier (2, 4, and 6 years prior to the measurement of wealth). Regressions control for a full set of covariates, including year and Census division fixed effects. *** *p* < 0.01; ** *p* < 0.05; * *p* < 0.10

The results show the evidence of both (a) asset depletion to meet immediate financial needs and (b) risk hedging to cope with the risk of recurrent cancer. Three results stand out. First, the cost of cancer was financed out of savings held in risky assets (*β*_0_ =  − 0.170, 95% CI: − 0.328 to − 0.012, *P* < 0.05), miscellaneous saving (*β*_0_ =  − 0.163, 95% CI: − 0.311 to − 0.016, *P* < 0.05), real estate equity (*β*_0_ =  − 0.167, 95% CI: − 0.298 to − 0.035, *P* < 0.05), and business equity (*β*_1_ =  − 0.145, 95% CI: −0.265 to −0.025, *P* < 0.05; and *β*_2_ =  − 0.175, 95% CI: −0.297 to −0.052, *P* < 0.01). The onset of diagnosis was associated with immediate liquidation of risky assets, miscellaneous saving, and real estate equity, and was followed by an additional decrease in risky assets and business equity through the continuing phase. Second, cancer survivors held additional liquidity after the treatment (*β*_1_ = 0.247, 95% CI: 0.126 to 0.367, *P* < 0.01; *β*_2_ = 0.184, 95% CI: 0.051 to 0.317, *P* < 0.01; and *β*_3_ = 0.159, 95% CI: 0.017 to 0.301, *P* < 0.05). The marginal effect interpretation and back-of-the-envelope calculation using sample means show that household savings held in cash and cash-equivalents increased by 17.2–28.0%, or by $8615–$14,005 from year + 2 to year + 8 since diagnosis. Given the magnitude of these estimates compared to those on risky assets and business equity, it is plausible to say that a portion of liquidated assets not spent on cancer care has been held as additional cash. Third, cancer accompanies a large increase in unsecured debts. The coefficient estimates on cancer range from 0.170 to 0.282 and reject the null hypothesis of non-significance at the 5% level across all post-cancer periods. Our rough estimate of additional liability due to cancer is $1314 in the acute phase, and is in the range of $915–$1609 for the continuing phase. The debt load peaks around year + 2 through year + 4 and remains significant throughout the post-cancer periods. Although there is no way to confirm if these estimates came from refinancing of the existing loan or the initial loan carried into the next periods, they all point to the lasting effects of cancer on household liabilities.

The relatively novel finding is that cancer patients reallocate their wealth from risky assets to the liquid form of savings. The primary motivation of this adjustment might be that insurance coverage does not provide enough financial protection against catastrophic medical expenditures. If health insurance fully indemnifies expected medical expenditures, cancer survivors would not show cash-stocking behaviors after diagnosis. To test for this argument, we examine if health insurance coverage moderates the association between cancer onset and household assets. Our empirical strategy is to interact cancer indicators with a dummy for having multiple coverages or employer-provided insurance (1 = multiple health insurance coverages or employer-provided plan; 0 = no health insurance or single coverage including government-provided insurance) and examine linear restrictions on the interaction terms. Table 5 presents the related results. Overall, our estimates show the evidence of asset depletion and risk hedging through portfolio relocation towards cash in both groups.

**Table 5:** Cancer effects on household wealth, by asset categories and insurance coverage (*N* = 68,634)

Response:	Log (cash)	Log (stocks+bonds)	Log (miscellaneous)	Log (retirement)	Log (housing)	Log (vehicles)	Log (estate)	Log (business)	Log (unsecured debt)
	(1)	(2)	(3)	(4)	(5)	(6)	(7)	(8)	(9)
*α*: Multiple insurance	0.380***	0.138**	0.040	0.075	0.414***	0.043	0.056	0.085	−0.060
	(0.065)	(0.061)	(0.059)	(0.068)	(0.097)	(0.058)	(0.060)	(0.053)	(0.078)
*β*_0_: Cancer *t*	−0.098	− 0.074	− 0.190**	0.015	−0.160	0.017	−0.100	0.006	0.180
	(0.087)	(0.104)	(0.093)	(0.097)	(0.128)	(0.078)	(0.089)	(0.071)	(0.110)
*β*_1_: Cancer *t* − 1	0.214**	−0.428***	0.033	−0.055	− 0.089	0.009	− 0.046	− 0.016	0.419***
	(0.089)	(0.115)	(0.108)	(0.110)	(0.142)	(0.089)	(0.103)	(0.083)	(0.118)
*β*_2_: Cancer *t* − 2	0.058	−0.349***	0.085	−0.021	0.107	−0.119	0.142	−0.222***	0.229*
	(0.101)	(0.121)	(0.117)	(0.122)	(0.138)	(0.092)	(0.105)	(0.084)	(0.120)
*β*_3_: Cancer *t* − 3	0.161	−0.055	− 0.158	− 0.146	0.148	0.140	−0.048	− 0.102	0.189
	(0.102)	(0.121)	(0.106)	(0.128)	(0.127)	(0.093)	(0.098)	(0.080)	(0.117)
*γ*_0_: *α* × *β*_0_	0.150	−0.195	0.054	− 0.031	0.197	− 0.001	− 0.134	− 0.170*	0.113
	(0.109)	(0.150)	(0.138)	(0.134)	(0.165)	(0.098)	(0.123)	(0.102)	(0.143)
*γ*_1_: *α* × *β*_1_	0.060	0.108	−0.060	0.141	0.032	0.004	−0.050	−0.250**	− 0.259*
	(0.109)	(0.157)	(0.159)	(0.144)	(0.180)	(0.108)	(0.137)	(0.109)	(0.148)
*γ*_2_: *α* × *β*_2_	0.237*	−0.098	− 0.190	− 0.101	− 0.291*	0.254**	− 0.252*	0.082	− 0.108
	(0.123)	(0.165)	(0.165)	(0.161)	(0.172)	(0.111)	(0.143)	(0.112)	(0.149)
*γ*_3_: *α* × *β*_3_	−0.001	− 0.007	− 0.034	0.232	− 0.419**	− 0.032	− 0.135	0.174	−0.038
	(0.133)	(0.168)	(0.165)	(0.171)	(0.170)	(0.120)	(0.140)	(0.115)	(0.151)
Linear restrictions:									
H_0_: *β*_0_ + *γ*_0_ =0	0.052	−0.269**	−0.136	− 0.016	0.037	0.016	−0.234**	− 0.164**	0.293***
H_0_: *β*_1_ + *γ*_1_ =0	0.274***	−0.320***	− 0.027	0.086	−0.057	0.013	−0.096	− 0.266***	0.160
H_0_: *β*_2_ + *γ*_2_ =0	0.295***	−0.447***	− 0.105	− 0.122	−0.184	0.135*	−0.110	− 0.140*	0.121
H_0_: *β*_3_ + *γ*_3_ =0	0.160*	−0.062	−0.192	0.086	−0.271**	0.108	−0.183*	0.072	0.151

Robust standard errors in parentheses. Subscript *t*-1, *t*-2, and *t*-3 represent variables measured in one, two, and three survey periods earlier (2, 4, and 6 years prior to the measurement of wealth). Regressions control for a full set of covariates, including year and Census division fixed effects. *** *p* < 0.01; ** *p* < 0.05; * *p* < 0.10

## Discussion

This study showed that household assets serve as a financial buffer against cancer care expenses as well as a higher financial risk associated with cancer recurrence. We found that cancer diagnosis was followed by significant reductions in investment asset, miscellaneous saving, real estate equity, and business equity, and modest increases in cash and cash-equivalent assets and unsecured debt, which is equivalent to an average of $125,832 (in 2015 dollars) net reduction in total net worth. We also found that additional cash holding emerged 2 years after the diagnosis and remained significant until the end of our tracking periods (year + 8 since the diagnosis). A similar pattern of asset depletion was observed for groups with limited health insurance coverage as well as those having multiple health insurance. Overall, cancer patients in our study exhibit portfolio reallocation towards low-risk and liquid assets and the evidence of precautionary saving in periods during which the risk of cancer recurrence is high (year + 2 through year + 8).

Our results show three notable findings. First, our estimate of asset depletion due to cancer was significantly larger than the previous estimates of OOP spending on cancer care. This difference can be explained by the fact that household wealth is a more inclusive measure of financial cost, which captures both direct costs and indirect economic burden on family members who would have forgone productive activities for caregiving. Our finding then indicates that using direct spending on cancer care would vastly understate the economic burden of cancer on patients and their family members.

Second, cancer survivors reallocated their savings portfolio away from risky equities and towards liquid assets that carry little or no financial risk. We found that cancer survivors held much less in risky assets, such as stocks and bonds, real estate equity, and business equity, but carried additional cash throughout the continuing phase of treatment. This pattern of portfolio reallocation was consistent with the background risky theory that increases in background risk offset willingness to bear other forms of risk [23]. Background risk is uninsurable, independent from financial risk, and not avoidable through diversification of asset portfolio [54, 55]. Heath risk is considered to be a typical example of background risk [56]. The onset of cancer is largely unpredictable and beyond one’s control; all else being equal, cancer patients face a higher risk of catastrophic medical spending than those in good health. Knowing the financial toxicity of cancer care from their experiences with an initial occurrence, cancer survivors have an incentive to reduce exposure to financial risk and accumulate additional liquidity to safeguard against the expected financial burden when tumor returns. A recent longitudinal study of American elderly reported a similar coping strategy that associates cancer with a draw-down of risky assets and increases in an emergency fund [57].

Third, having additional health insurance had a limited impact on the extent to which households with cancer patients deplete their wealth and using cash as a financial buffer. The substitution between financial risk and health risk is more pronounced for a sample we believe to be well-insured (through either employer-provided plan or multiple health insurance coverages not including Medicare + Medicare), compared with a less insured group (single coverage or coverages only from public insurance). These findings suggest that insurance policies that cover only the costs of medical care are likely to provide only partial protection against the full cost of cancer. As noted in the cost estimates, current public and private health insurance programs are insufficient to fully prevent financial distress resulting from various forms of indirect costs.

### Limitations

This study has a number of limitations. First, our analysis is subject to survivorship bias resulting from mortality differential across pathologic stages. The lower life expectancy for toxic tumors raises the concern that patients with advanced cancer or certain types that have low survival rates are under-represented in the sample and those with curable types are over-represented. Since treatment costs are higher for advanced cancers [58], our estimates of wealth effect might be biased downwards. Second, the cancer screening question in the HRS excludes skin cancer and non-malignant tumors. It is possible that those with skin cancer or non-malignant tumors were assigned to the non-cancer group, and this measurement error led to a smaller difference in wealth by cancer history. Third, the estimates could be confounded by voluntary changes in consumption behaviors. The onset of cancer could have reduced non-health consumption by limiting mobility, travel opportunities, and the ability to consume food away from home. At the same time, it could motivate patients to consume “before it’s too late” and results in a higher rate of spending in anticipation of survival risk [26]. These two forces can either amplify or weaken cancer-wealth correlation depending on which channel carries a larger effect. Fourth, debt accumulation post-cancer could be the manifestations of strategic considerations by patients. For instance, patients with only a few years to live may increase debts and choose to strategically default on them [59]. If then, changes in liabilities are determined by catastrophic medical expenses as well as a lack of intention to repay debts. Due to the limitations in data, we are unable to test this motive and its impact on the estimated cancer effect.

## Conclusions

Our cost estimate suggests that middle-aged and elderly Americans need to take into account the potential medical costs associated with cancer onset in retirement planning. An important implication of wealth depletion following a cancer diagnosis is that senior households are not holding enough financial buffer to cope with medical expenses. The onset of cancer can be particularly detrimental to older adults due to limited income sources after retirement and a higher risk of complications associated with cancer treatment. In particular, patients with more toxic cancers are at higher risk for costly health and financial consequences. Financial advisors and practitioners are encouraged to use our findings to advise clients with a family cancer history on how much emergency saving to hold and how to allocate household assets.

Our finding on the depletion pattern across insurance coverage supports further expansion of Medicare. We observed reallocation of household wealth towards liquid assets for both well-insured and under-insured groups, implying that cancer patients found financial protection through health insurance inadequate. These results are consistent with the recent finding that a third of insured cancer patients pay more expenses out-of-pocket than they expected, despite having health coverage [60]. Medicare program needs to pick up more co-payments associated with curative care and expand coverage to home-based long term care and palliative care, which are not included in the current benefit category. Besides, individuals with a family history of toxic tumors may want to consider a private cancer insurance policy to offset the anticipated financial consequence of cancer diagnosis.

Given the rising cost of new cancer therapies, there has been considerable interest in the relationship between cancer-related treatment expenses and mortality outcomes post-treatment [11]. A study of the 1996–2004 HRS estimated that older persons with one or more financial hardships have 1.4–1.8 times higher hazard ratios than those without financial difficulties [61]. The financial distress from mounting financial obligations and debt is a major risk factor for mortality after cancer diagnosis [62]. The depletion of wealth may come with greater emotional distress for older patients due to limited labor income after retirement. A possible direction of future research is to examine how asset depletion for cancer care affects mortality risk and quality of life among cancer survivors.

## Data Availability

The datasets of the current study are publicly available at the Health and Retirement Study data portal (https://hrs.isr.umich.edu/data-products/access-to-public-data). The computer codes that convert the original data to estimates are available from the corresponding author on reasonable request.

## Abbreviations

CPI-U: Consumer price index for all urban consumers (CPI-U)
HRS: Health and Retirement Study
MEPS: Medical Expenditure Panel Survey
OOP: Out-of-pocket

## Footnotes

1 The preliminary analysis shows that the effect of cancer on wealth does not persist beyond three survey periods. Therefore, the maximum lag length of cancer is three.
